# Surface Texture Designs to Improve the Core–Veneer Bond Strength of Zirconia Restorations Using Digital Light Processing

**DOI:** 10.3390/ma16186072

**Published:** 2023-09-05

**Authors:** Kang Dai, Jiang Wu, Zhen Zhao, Hai Yu, Zhe Zhao, Bo Gao

**Affiliations:** 1 State Key Laboratory of Military Stomatology & National Clinical Research Centre for Oral Diseases & Shaanxi Key Laboratory of Stomatology, Department of Prosthodontics, School of Stomatology, The Fourth Military Medical University, Xi’an 710032, China; 2 School of Material Science and Engineering, Shanghai Institute of Technology, Shanghai 201418, China

**Keywords:** digital light processing (DLP), zirconia, veneer, shear bond strength (SBS), surface textures

## Abstract

Veneered zirconia ceramics are widely used for dental restorations. However, the relatively poor bonding strength between the ceramic core and veneer porcelain remains a common problem in clinical applications. To address this issue, this study focused on enhancing the core–veneer bond strength of zirconia restorations through the implementation of surface textures using digital light processing (DLP) technology. The light intensity was precisely tuned to optimize mechanical strength and minimize light scattering. Subsequently, hexagonal or square grids were printed on the surface of the zirconia ceramic core. Following veneering procedures, the shear bond strength (SBS) test was conducted using a universal testing machine. Dates were compared using analysis of variance (ANOVA) and the least significant difference (LSD) test. Furthermore, optical microscopy and scanning electron microscopy (SEM) were used to examine the failure modes and observe the cross-sectional structures, respectively. The results indicated that the presence of a 0.09 mm high hexagon grid led to a significant 21% increase in the SBS value. However, grids with heights of 0.2 and 0.3 mm showed less improvement, owing to the formation of large defects at the interface during the fusion process. This study demonstrated the potential of DLP technology in preparing zirconia ceramics with complex structures and high mechanical strength, thereby offering promising solutions for overcoming challenges associated with dental applications.

## 1. Introduction

Zirconia has attracted great attention as a dental restorative material, due to its high mechanical strength, steady chemical properties, and excellent biocompatibility [1]. However, to achieve optimal esthetics, zirconia is typically layered with translucent veneering porcelain, due to its opaque appearance. Clinical research and practice have revealed that, although zirconia frameworks are extremely resistant to fracture, delamination of the veneering ceramics is still a common problem [2]. Notably, veneer fracture incidences in zirconia fixed dental prostheses is remarkably higher than those in metal–ceramic fixed dental prostheses [3]. The success of veneered zirconia restorations over an extended period is limited by the poor performance of the veneering ceramics and their weak bonding to the zirconia core. Such weak bonding is related to several factors, including lack of chemical bonding, a disparity in thermal expansion coefficients, poor wetting of veneering on the core, etc. [4,5]. Therefore, various surface modification techniques were developed to increase the roughness, contact area, and surface energy of zirconia.

Sandblasting is the most frequently used method to increase bond strength [6]. However, sandblasting may introduce flaws and defects that are detrimental to the mechanical properties of zirconia [7]. Laser irradiation is another popular surface modification technique in dentistry. Surface particles can be removed via ablation and vaporization during laser processing, resulting in surface textures with uniform geometry, as opposed to random surface roughness. Well-designed dimples, grooves, and grids can be produced with this treatment on zirconia ceramic [8]. Arami et al. [9] reported that surfaces of zirconia treated using an Er:YAG laser showed roughness similar to that achieved with air abrasion treatments. Henriques et al. [10] evaluated the impact of Nd: YAG laser surface structuring on the bond strength of veneering porcelain to zirconia. Equally spaced micro-holes were created using a laser on the surface. The results indicated that laser structuring could greatly increase shear bond strength. In another study, specimens were grooved with line scans using a femto laser. The size of the grooves macroscopically increases the bonding area, and seems advantageous compared to sandblast treatments [11]. Akay et al. [12] employed an ultrafast UV laser for the processing of Y-TZP ceramic and produced various surface textures, such as grids, holes, and grooves. All of the textured samples exhibited remarkably higher flexural bond strength in comparison to the nontreated samples.

Although the results of laser texturing to enhance bond strength are promising, it also has some disadvantages, including thermal cracking and laser-induced phase transformation [13]. Both of these consequences could weaken the ceramic’s strength and impair the stability of zirconia restorations [14]. In addition, the laser beam direction for laser surface texturing should typically be perpendicular to the surface. However, if the geometry of the restoration is complex, it may be difficult to fulfill this requirement. As a result, reproducing surfaces with consistent textures is challenging, which reduces the efficiency of the designed function [15].

Recently, additive manufacturing (AM) technology has emerged in the field of dentistry. AM builds up a structure by adding materials layer upon layer. The aforementioned procedure facilitates the creation of a vast array of geometric designs and enables the manufacturing of numerous customized components simultaneously. Hence, complicated structures that are unattainable through subtractive techniques using milling tools can be conveniently produced through AM. Among the AM technologies, the digital light processing (DLP) technique exhibits the capacity to generate ceramic components possessing exceptional precision and favorable mechanical characteristics [16]. Rungrojwittayakul et al. [17] reported that the trueness of dental models was related to printer technology, and they proved that DLP technology achieved clinically acceptable accuracy. Therefore, in this study, we intend to investigate the effects of surface textures created using DLP technology on the core–veneer bond strength of zirconia restorations. The null hypothesis was that core–veneer bond strength would not be improved by the surface textures obtained using DLP technology. To this end, various surface textures were printed and evaluated. In addition, studies were conducted to optimize the flexural strength and geometry precision of the DLP-printed zirconia ceramics, to provide a strong foundation for this research.

## 2. Materials and Methods

### 2.1. Slurry and Green Body Preparation

In total, 3 mol% yttria-stabilized zirconia (CP-TZP-010, Jiaxing CeramPlus Technology Co., Ltd., Jiaxing, China) was used as it was received. For the preparation of the slurry, with a solid loading of 50 vol%, the powders were mixed with a commercially available photosensitive resin (11.5 wt.% base on the slurry, Jiaxing CeramPlus Technology Co., Ltd., Jiaxing, China). 0.2 wt.% Diphenyl (2,4,6-trimethylbenzoyl) phosphine oxide (Shanghai Dibo Chemical, Shanghai, China), which has an efficient absorption peak between 350 and 410 nm, was employed as a photoinitiator. 

A top-down DLP printer (DLP-Desktop, Jiaxing CeramPlus Technology Co., Ltd.) was utilized to load the suspension and cure the resin, using UV light with a wavelength of 385 nm. The DLP printer exhibited a resolution of 50 μm in the *x*–*y* plane. SolidWorks 2021 was utilized to generate models in the STL file format. A 35 mm × 4 mm × 3 mm model was created for the flexural strength test. The printing layer thickness and exposure time were set as 30 μm and 4 s, respectively. Following the printing procedure, excess slurry was eliminated from the samples through the utilization of ethanol.

### 2.2. Thermal Analysis

To investigate the pyrolysis of photosensitive resin, thermogravimetry–differential scanning calorimetry analysis (TG-DSC, Q600, TA Instruments, New Castle, DE, USA) was performed under air atmosphere from 20 °C to 700 °C, with a 5 °C/min heating rate, followed by the design of a suitable debinding process.

### 2.3. Measuring Geometrical Overgrowth

Light-scattering effects widened the cured area of the slurry, which could influence printing accuracy. To investigate the impact of the exposure dosage on the overgrowth of the slurry, a grid model, with a length of 0.62 mm and wall thickness of 0.25 mm, was designed and printed with different exposure energies. The researchers utilized the KEYENCE Image Dimension Measurement System (LM-1000, KEYENCE, Osaka, Japan) to assess the accuracy of the printing. 

### 2.4. Flexural Strength Tests

To investigate the impact of exposure dosage on the mechanical properties of ceramics, bending bar samples fabricated using different levels of exposure energy were evaluated for their flexural strength. For each group, 10 sintered samples were tested, using a three-point bending tester (WD-03, Shanghai ZhuoJi, Shanghai, China) with a 24 mm span and a 0.5 mm/min loading rate [18,19].

### 2.5. Characterization

The densities of the zirconia ceramics were measured using the Archimedes method (YDK03, Sartorius, Gottingen, Germany). The microstructure of the DLP-printed zirconia ceramics was analyzed using a SEM (Sigma 300, Zeiss, Oberkochen, Germany).

### 2.6. Shear Bond Strength (SBS) Test

#### 2.6.1. Specimen Preparation

**Figure 1:**
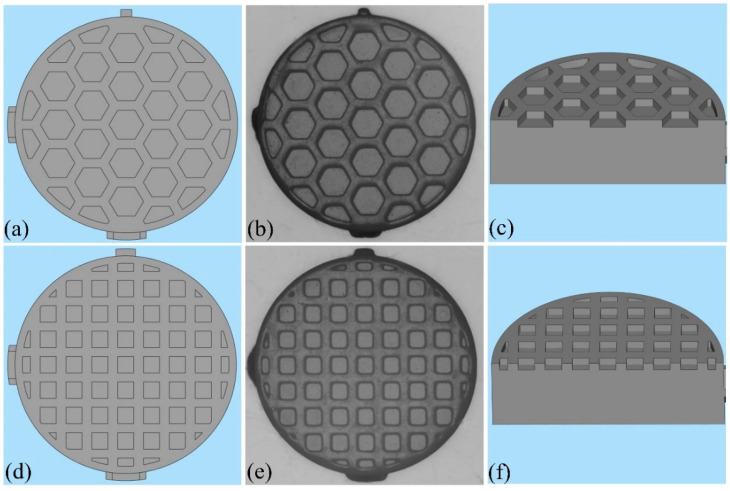
Computer-aided designs and corresponding sintered structures: (**a**,**d**) top view of the computer-aided designs; (**b**,**e**) sintered structures; (**c**,**f**) cross section of the computer-aided designs.

In total, 140 zirconia specimens, fabricated using DLP technology with optimized parameters, were divided into 7 groups (*n* = 20) according to the grids in the sample surface. The cylindrical samples (which had thicknesses of 2 mm and diameters of 6 mm) had two kinds of surface designs: one with a hexagon grid and the other with a square grid (Figure 1). Specimens without grids served as the control group.

The side length of the grids, as designed, was 0.4 mm. The printing layer thickness was 30 μm, and the linear shrinkage in the building direction was 78%. Thus, the thickness of each sintered layer was 0.023 mm. Overall, 4, 9, and 13 layers were selected with corresponding grid depths of 0.092, 0.207, and 0.299 mm, respectively. The study comprised 7 groups: group 1, with no grids on the surface (control group); group 2, a regular hexagon grid with a depth of 0.09 mm; group 3, a regular hexagon grid with a depth of 0.20 mm; group 4, a regular hexagon grid with a depth of 0.29 mm; group 5, a quadrilateral grid with a depth of 0.09 mm; group 6, a quadrilateral grid with a depth of 0.20 mm; and group 7, a quadrilateral grid with a depth of 0.29 mm. The KEYENCE Image Dimension Measurement System was used to evaluate grid accuracy.

#### 2.6.2. Veneering Procedures and the SBS Test

**Figure 2:**
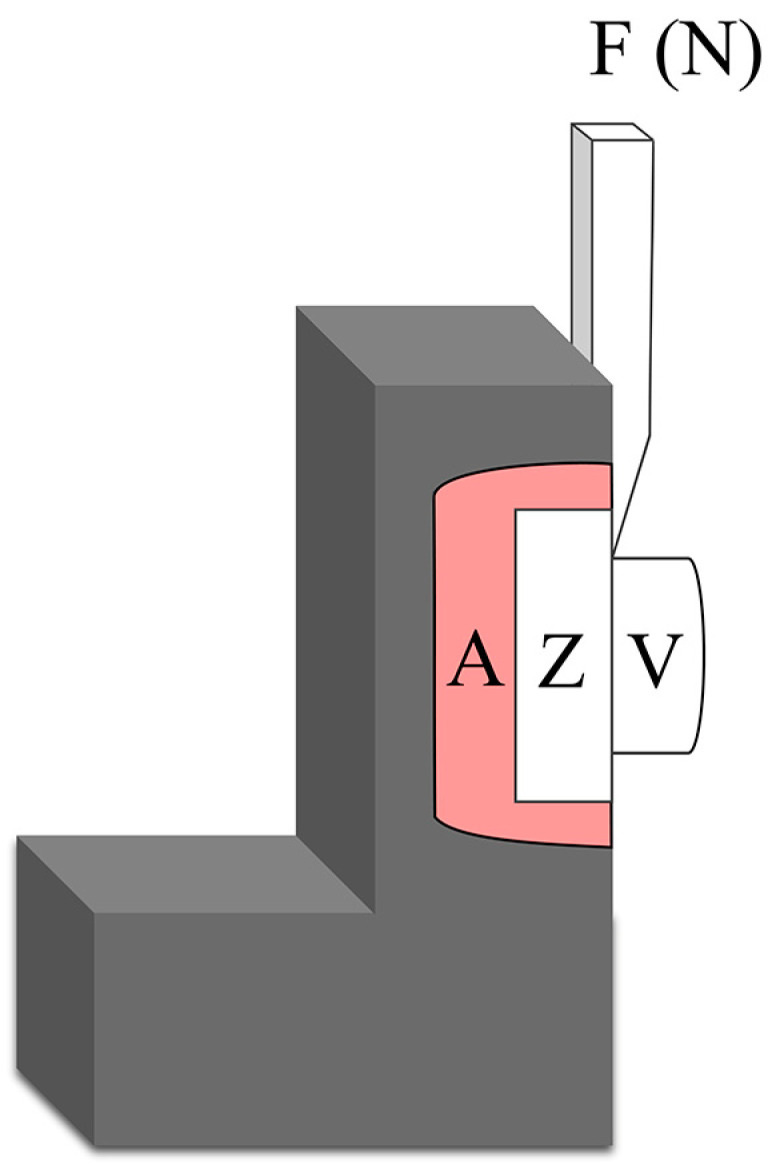
Schematic diagram of the shear bond test in the universal test. A—acrylic resin; Z—zirconia ceramic; V—veneering ceramic; F—shear force.

The manual layering method was employed to carry out the veneering process. Initially, a liner material (IPS e.max Ceram ZirLiner, Ivoclar, Schaan, Liechtenstein) was applied and subsequently subjected to independent firing. Thereafter, veneering porcelain IPS (IPS e.max Ceram, Ivoclar, Schaan, Liechtenstein) was built up to all specimens at 2 mm in height and 4 mm in diameter using a mold. After vibration, the specimens were fired in the furnace (Programat P300, Ivoclar, Schaan, Liechtenstein) following the manufacturer’s recommendations. Due to shrinkage after firing, the specimens’ exact veneer layer diameter was measured using a vernier caliper (Guilin GuangLu, Guilin, China). Using self-curing acrylic resin (Shanghai Medical Instruments Co., Ltd., Shanghai, China), each specimen was secured in a metal container in a universal testing device (Figure 2). To delaminate the veneering porcelain, the load was delivered using a wedge, at a crosshead speed of 0.5 mm/min, while maintaining parallel alignment with the specimen’s bonding surface. After that, the shear bond strength (MPa) was estimated by dividing the failure load (N) by the interface area (mm^2^) [20,21]. 

**Figure 3:**
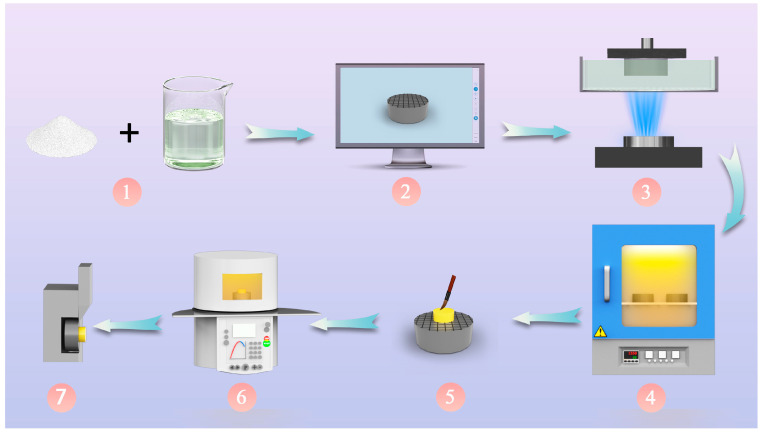
Flowchart of this study: 1—suspension preparation, 2—model design, 3—printing, 4—debinding and sintering processes, 5, 6—veneering procedures, and 7—SBS test.

The SEM was utilized to observe the cross-sectional structure. Optical microscopy was employed to examine the fracture surfaces, and the failure modes were recorded and defined as adhesive failure (failure at the zirconia surface), cohesive failure (failure within the veneer or zirconia), or mixed mode (combination of cohesive and adhesive failure modes) [22,23]. In addition, representative samples were examined using a SEM. A flowchart of the experiment is presented in Figure 3.

## 3. Results 

### 3.1. Thermal Analysis and Thermal Treatment

**Figure 4:**
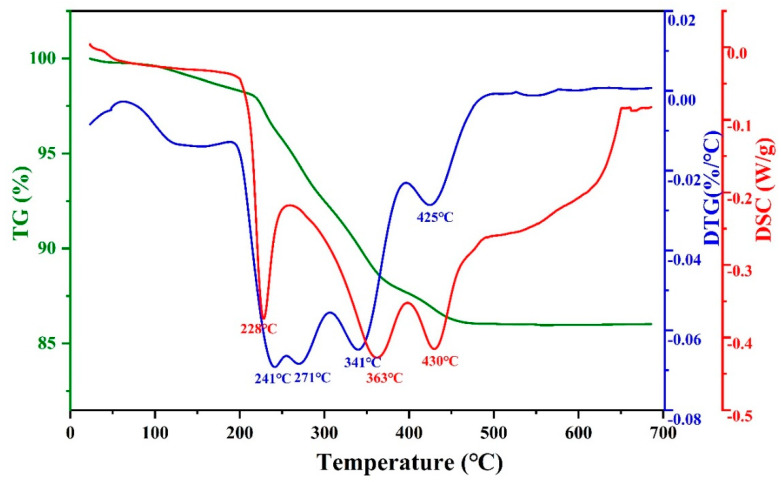
TG−DSC curves of the green body.

The TG−DSC curves are presented in Figure 4. The TG curves indicate that the process of organic decomposition is initiated at a temperature of 100 °C. A flattening in the TG curve at over 500 °C suggests that all organic material has been decomposed. In the derivative thermogravimetry (DTG) curve, there were four decomposition peaks: 241 °C, 271 °C, 341 °C, and 425 °C. During the debinding process, it was observed that the resin underwent rapid decomposition within the temperature range from 241 °C to 425 °C. The DSC curve showed three exothermic peaks at 228 °C, 363 °C, and 430 °C.

**Figure 5:**
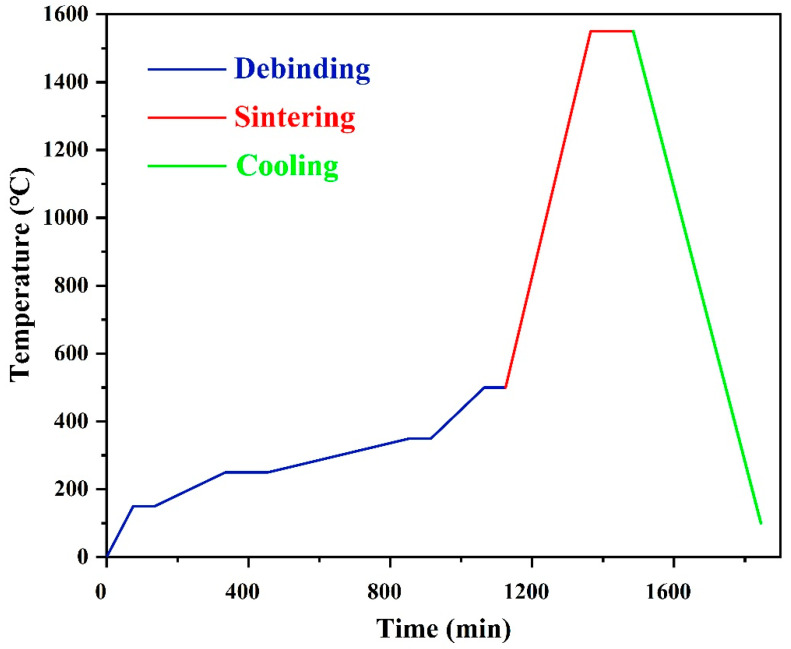
Scheme of the debinding and sintering processes.

The processes of debinding and sintering are depicted in Figure 5. To avoid the exacerbation of defects in the debinding stage due to the rapidly expanding gas, the rate of temperature rises between 100 °C and 500 °C was carefully controlled [18]. Four distinct heating rates of 2, 0.5, 0.25, and 1 °C/min were employed for the debinding profiles, each corresponding to specific temperature ranges of RT–150 °C, 150–250 °C, 250–350 °C, and 350–500 °C, respectively. The dwelling time at temperatures of 150 °C, 350 °C, and 500 °C was 60 min, which was doubled at 250 °C. After performing the debinding process up to 500 °C, sintering was performed with a 5 °C/min heating rate up to 1550 °C, which was then held for 120 min.

### 3.2. Geometrical Overgrowth

**Figure 6:**
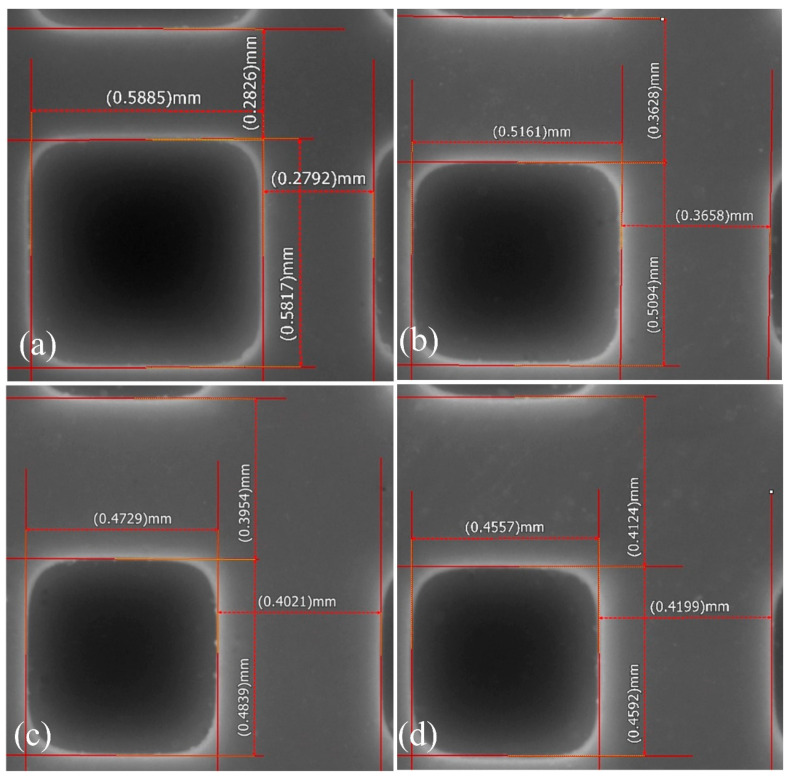
Measurements of printed samples with increasing exposure dose. The light intensities utilized were (**a**) 8 mW/cm^2^, (**b**) 18 mW/cm^2^, (**c**) 28 mW/cm^2^, and (**d**) 36 mW/cm^2^.

The findings of the overgrowth tests are displayed on the printed green body in Figure 6. This figure brings up the fact that, with increased light intensity, the wall thickness of the grid increased, from 0.28 to 0.42 mm. Meanwhile, the grid length decreased, from 0.59 to 0.46 mm. From the resultant picture, we can observe that the exposure energy applied had a strong influence on the dimensional accuracy. The extent of the overgrowth grew markedly as the dose of exposure increased, resulting in significantly extended light scattering. Based on these results, the subsequent printing process should use a lower exposure dose, which would lead to a remarkable improvement in printing accuracy. At 8 mW/cm^2^, the single-edge light scattering-induced overgrowth was only 0.015 mm (Figure 6a), which is regarded as a good printing accuracy. When the light intensity was more than doubled, to 18 mW/cm^2^, the overgrowth increased to approximately 0.058 mm. This result indicates that the best exposure intensity should be around 8 mW/cm^2^.

### 3.3. Flexural Strength of Ceramics with Different Exposure Energies

In addition to the accuracy of the printing, the mechanical strength should also be taken into account. The light intensity levels were set at 4, 6, 8, and 10 mW/cm^2^. Table 1 displays the bending strength of samples made using these various exposure energy levels. Four data groups of flexural strength were found to be of uneven variance (*p* = 0.007). The mean strength of the groups was compared using Welch’s test. Multiple comparisons were made using Tamhane’s T2 test. The flexural strength of group III complied with the ISO6872:2015 [24] standard (Dentistry: ceramic materials, flexural strength of four or more units of prosthesis >800 MPa) and was statistically higher than those of the other three groups. Moreover, because of insufficient exposure energy, the green body of group I was still very soft, which makes post-printing handling difficult. The light intensity was determined at 8 mW/cm^2^ to ensure the mechanical characteristics and sample accuracy, and this resulted in a curing depth of 58 μm (nearly twice as thick as the layer thickness), which was anticipated to enable enough bonding between the interlayers.

### 3.4. Microstructure of DLP-Printed Zirconia Ceramics

**Figure 7:**
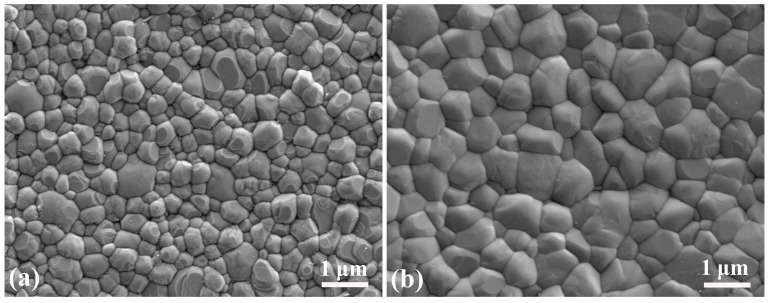
SEM secondary electron images of zirconia ceramics (30,000×): (**a**) DLP-printed zirconia ceramics and (**b**) milled zirconia ceramics.

Figure 7a shows that the DLP-printed zirconia ceramic fabricated with optimized parameters possesses a dense microstructure. The zirconia grains aggregated tightly, and the average diameter was approximately 400 nm. Upon undergoing sintering at a temperature of 1550 °C, the zirconia ceramic exhibited a density of 6.02 g/cm^3^, indicating a relative density of 99.2%. Figure 7b shows the dense microstructure of the milled zirconia ceramic (Zenostar, Wieland Dental, Pforzheim, Germany). A compact microstructure and fine grains are crucial for achieving elevated density and superior mechanical characteristics in zirconia ceramics [25]. 

### 3.5. SBS Test

#### 3.5.1. Measurements of the Grids

**Figure 8:**
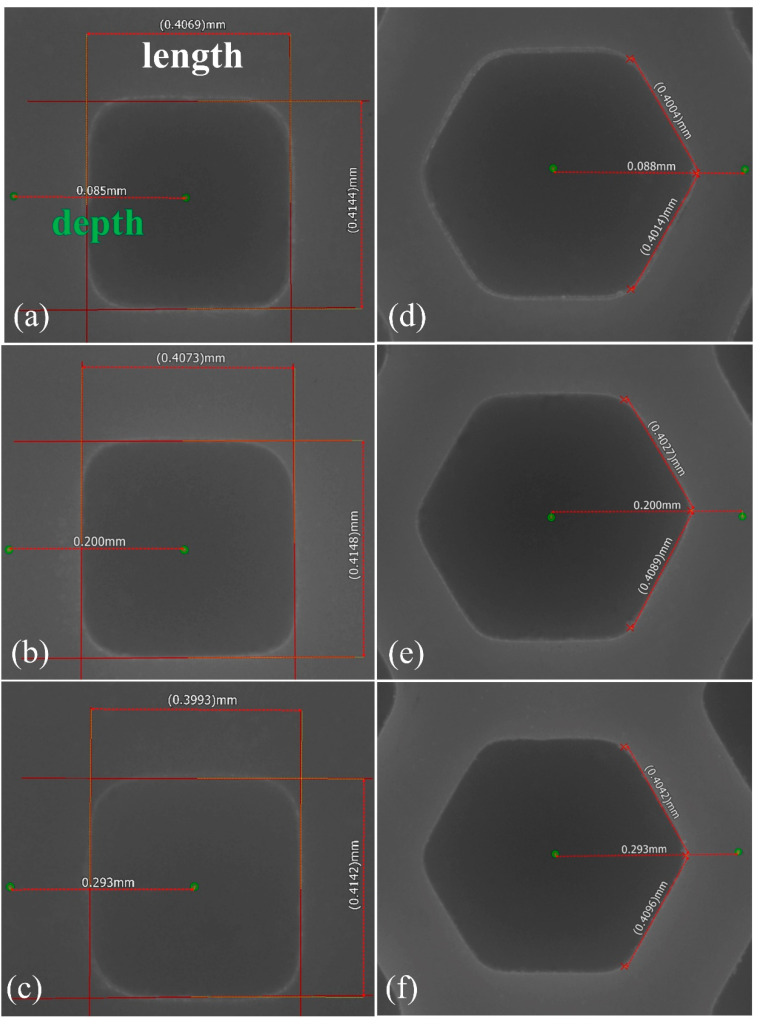
Grid size measurements for the designed depth and length of the square grids, (**a**) 0.092 mm and 0.4 mm, (**b**) 0.207 mm and 0.4 mm, (**c**) 0.299 mm and 0.4 mm, and of the regular hexagon grids, (**d**) 0.092 mm and 0.4 mm, (**e**) 0.207 mm and 0.4 mm, and (**f**) 0.299 mm and 0.4 mm.

Figure 8 shows the measured results of the grids. Values between two green points represent depth. The designed grid depths were 0.092, 0.207, and 0.299 mm, and the measured depths were 0.085, 0.200, and 0.293 mm, respectively. The measured grid lengths ranged from 0.3993 to 0.4148 mm, values which were very close to the designed length of 0.4 mm. It can be seen that the size precision obtained using DLP qualifies well for dental application. 

#### 3.5.2. SBS Values of the Test Groups

Table 2 presents the SBS values. The data were analyzed using SPSS 20.0 (IBM Corp, Armonk, NY, USA). The effects of grid shape and depth were investigated using two-way analysis of variance (ANOVA). The LSD test was performed for multiple comparisons. Statistical results revealed a significant influence of depth on mean SBS values (*p* = 0.024), unlike grid shape (*p* = 0.1). No significant interactions were found between grid shape and depth (*p* = 0.267). The SBS values of group 2 (*p* = 0.022) and group 5 (*p* = 0.01) were significantly higher when compared to those of group 1. However, the SBS values of the other four experimental groups were not significantly different from those of group 1.

#### 3.5.3. SEM Secondary Electron Images of Cross-Sectional Structure 

**Figure 9:**
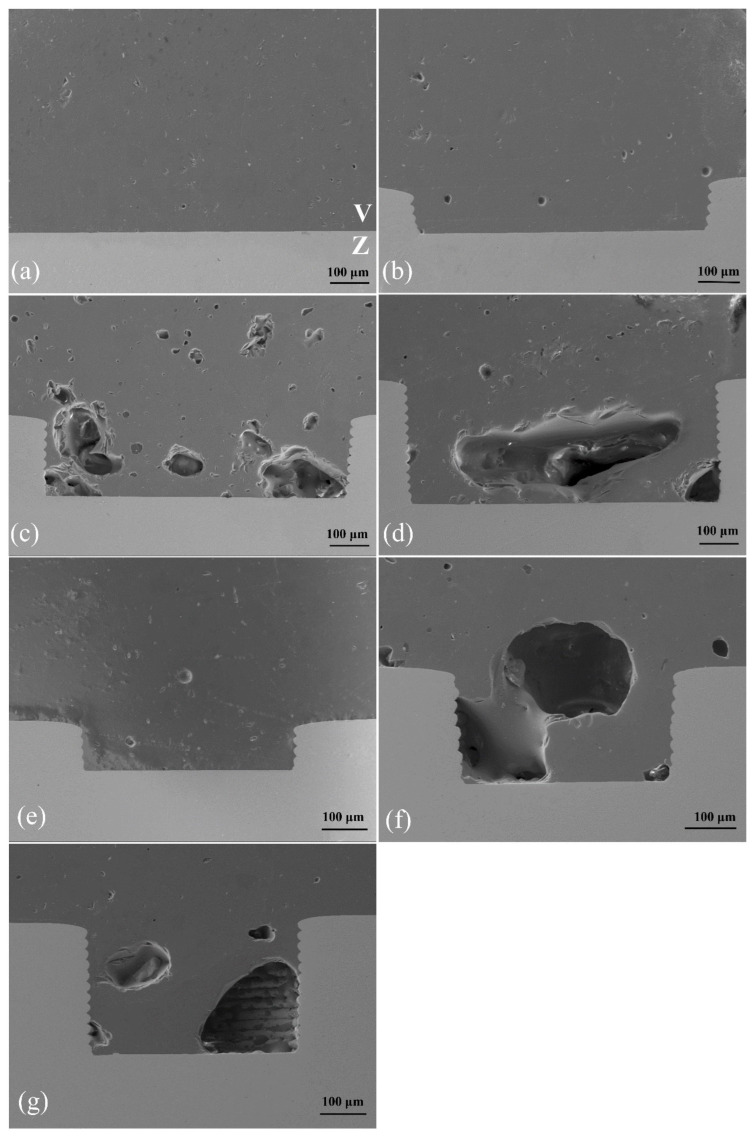
Cross-sectional morphology of the interface between veneering porcelain (V) and zirconia ceramic (Z), (300×): (**a**) group 1, (**b**) group 2, (**c**) group 3, (**d**) group 4, (**e**) group 5, (**f**) group 6, and (**g**) group 7. Obvious pores as structural defects were observed in (**c**,**d**,**f**,**g**).

As revealed in the SEM secondary electron images of the cross-sectional structure, the veneering porcelain infiltrated into the grids. Figure 9a,b,e demonstrate that the veneer ceramics were well attached to the zirconia ceramics. When the grid depth was >0.2 mm, structural defects were obvious within the veneer ceramics. Pores larger than 300 μm were observed, as shown in Figure 9c,d,f,g.

#### 3.5.4. Fracture Topography of Specimens with Different Surface Textures

**Figure 10:**
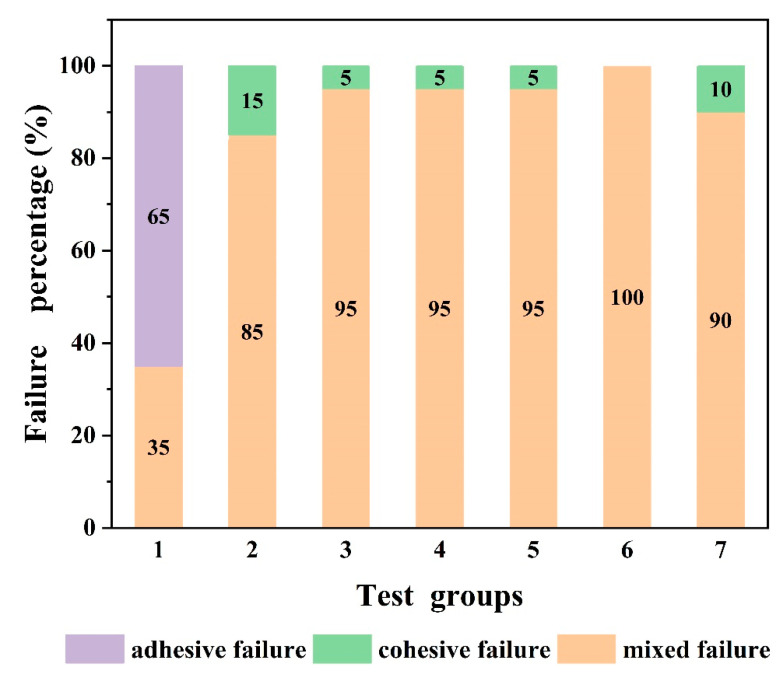
Failure mode percentage of test groups.

**Figure 11:**
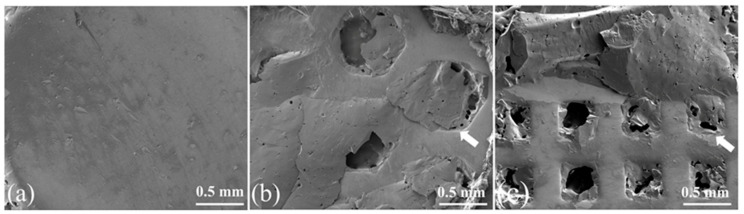
SEM secondary electron images of fractured zirconia surfaces (40×): specimens (**a**) group 1, without grids; (**b**) group 3, with hexagonal grids; and (**c**) group 6, with square grids. White arrows indicate residual porcelain retained in the grids and partially attached on the debonded zirconia surfaces.

Adhesive and cohesive failures were observed in the control group, while specimens in the experimental groups exhibited a higher percentage of mixed mode failure (Figure 10). Figure 11a revealed that almost none of the veneers remained attached to the zirconia, whereas Figure 11b,c showed larger parts of the veneering porcelain retained in the grids after the SBS test. The fractures were found to start at the veneer ceramic and in the interface between zirconia and veneer, except in the case of cohesive fractures that only occurred in the veneer. None of the zirconia grids were damaged.

## 4. Discussion

By optimizing the processing parameters, the designed zirconia samples were fabricated successfully. According to the findings of this investigation, the zirconia samples examined possessed both high levels of accuracy and favorable mechanical characteristics. This outcome could possibly be attributed to the suitable exposure energy that was selected properly. Insufficient exposure energy may result in inadequate bonding between the cured layers, leading to delamination. This subsequently leads to defects that can impair the mechanical strength of the ceramics. However, if the exposure energy is high, the slurry outside of the originally exposed area is partially cured, owing to the impact of light scattering. This, in turn, leads to poor accuracy [26]. For example, intricate details (e.g., irregular grooves, valleys, crannies) in dental restorations are difficult to form under high exposure energy. The light curing strategies used in this experiment achieved a balance between accuracy and mechanical strength.

In this study, the flexural strength of the DLP-printed samples reached 952 MPa, which was slightly lower than 978 MPa, which was reported by Wang et al. [19]. In their study, the demand for maximizing the flexural strength of zirconia ceramics manufactured using a stereolithography (SLA) process was achieved using the Taguchi method. However, the requirements for printing accuracy were ignored, and the adaptations in the SLA crown were not as good as those of the cutting crown, which may be due to unsuitable scanning parameters [27]. Accuracy and adaptation are closely related in dental crowns. A high dimensional accuracy is associated with improved clinical adaptation. Inadequate adaptation of the crown can lead to microleakage and plaque accumulation, thereby increasing the risk of debonding and gingival inflammation. Therefore, Zhang et al. [28,29] indicated a preference for soft-start light-curing strategies and a higher depth of cure values for the DLP system. Soft-start light-curing strategies decrease internal stresses originating from photopolymerization, whereas a higher cure depth improves the interlaminar bonding of the layers [30].

Surface texturing can potentially improve bond strength because it increases both the roughness and the bonding area [31]. In this study, the desired surface texture was achieved by printing grids on the surface of the zirconia ceramics. SEM analysis revealed that the molten veneering porcelain penetrated into the grids, generating an interlocking structure of veneer and zirconia at the interface. This structure provided interlocking bonding, which improved the SBS value through micromechanical interaction and chemical bonding [32].This could explain the higher SBS values observed in groups 2 and 5, as compared with the control group. Additionally, the fracture topography and higher percentage of mixed failure partly reflected the resultant strengthening effect. Thus, the research hypothesis was rejected. It is interesting that group 2 exhibited a higher percentage of cohesive failures than group 5. The hexagonal grid was larger than the square grid, and it had an obtuse internal angle (120°). This geometry may allow for easier infiltration of molten veneering porcelain into the hexagonal grids than into the square grids. Therefore, more cohesive failures were observed in group 2.

However, the SBS values of the other experimental groups showed less improvement. When veneer ceramic powder is mixed with the manufacturer’s liquid, air can enter the pore space of the porcelain slurry. The porcelain furnace could not attain a complete vacuum, due to its low vacuum degree (5 kPa). This would cause residual air to concentrate and form large bubbles during the sintering process [33]. These bubbles rose to the glass surface and released when the grid depth was 0.09 mm. However, the bubbles could not further diffuse with the increase in depth, which was related to the high viscosity of the porcelain melt. Therefore, large pores were generated within the veneer ceramics, which might weaken the interfacial bond [21,22,34]. A number of holes were observed in the veneering porcelain, and the reason for this lack of improvement might be the presence of defects in the veneer ceramics at the interface. Although the bubbles can be reduced by applying a higher sintering temperature [35], this method would cause flaws and cracks in the porcelain, and was prohibited by the manufacturer.

Previous investigations indicated that the SBS value of the IPS porcelain to zirconia ceramic ranged from 16.16 to 22.1 MPa [3,22,36,37]. The results obtained in this study were close to those reported in earlier experiments. However, some differences were noted between this study and prior research. First, in these earlier studies, airborne particle abrasion was applied before veneering. According to researchers, sandblasting has the potential to serve as a crucial element in the emergence of surface flaws and voids, thereby hindering the effectiveness and reliability of zirconia restorations [23]. In addition, sandblasting might result in phase transformation, from tetragonal to monoclinic (t → m), in the zirconia surface, which has an adverse influence on bond strength [20]. Thus, the latest instructions from IPS do not advise sandblasting. Other differences, including the type of zirconia material, the geometry of the specimens, and the rate of load application, will have effects on the experiment results. This study suggests that the SBS value of the porcelain to DLP-printed zirconia was in the same range as that of commercially available zirconia ceramics, and this value could be improved with printing grids. Compared with sandblasting and laser treatments for improving bond strength, the proposed method has some unique advantages, such as a desirable net shape and the nondestructive nature of the treatment.

The field of dentistry has a broad range of applications for AM techniques, including the production of crowns, inlays, bridges, surgical guides, implants, and orthodontic materials. Dentistry is one of the rapidly emerging markets for AM technologies, due to the increasing demand for patient-specific, custom-made dental products [38]. AM facilitates a transition from mass production to mass customization, resulting in a notable enhancement in productivity and a reduction in manufacturing expenses. The present study provided a promising result, showing that, in addition to the flexibility for realizing complex structures, AM technologies can solve practical problems. 

There are a few limitations of our study. Firstly, the utilized specimens did not represent the clinical restoration shape. Secondly, neither thermocycling nor mechanical aging procedures were applied in this study. These aspects should be considered in subsequent studies. Future research must also address other important aspects, such as the color stability, wear resistance, and biocompatibility of the DLP-printed zirconia ceramics [39].

## 5. Conclusions

Surface-textured zirconia ceramics with high precision can be successfully fabricated using DLP technology. The results indicated that a value of 8 mW/cm^2^ achieved the highest bending strength, of 952 MPa, and the lowest light scattering-induced overgrowth, of 0.015 mm. The shear bond strength of IPS porcelain to DLP-printed zirconia ceramics was improved by printing grids at a depth of 0.09 mm. Grids with a height > 0.2 mm showed comparatively lower effectiveness of such an improvement.

## Figures and Tables

**Table 1:** Summary of the flexural strength among the four groups.

Group	Light Intensity (mW/cm^2^)	Flexural Strength (MPa)
I	4	712 ± 79 ^a^
II	6	645 ± 148 ^a^
III	8	952 ± 75 ^b^
IV	10	668 ± 192 ^a^

Values marked with different lowercase letters indicate a significant difference among groups (*p* < 0.05).

**Table 2:** SBS values of DLP-printed zirconia ceramics with different surface textures.

Group	Description	SBS (MPa)
1	No grids in the surface	15.37 ± 3.45
2	Regular hexagon grids 0.09 mm deep	18.65 ± 3.95
3	Regular hexagon grids 0.20 mm deep	17.90 ± 4.32
4	Regular hexagon grids 0.29 mm deep	17.32 ± 4.48
5	Square grids 0.09 mm deep	18.99 ± 3.28
6	Square grids 0.20 mm deep	15.21 ± 3.75
7	Square grids 0.29 mm deep	15.91 ± 4.65

## Data Availability

All data are reported in the article.
